# Uterine Tumours

**Published:** 1901-12

**Authors:** 


**Uterine Tumours.** By W. Roger Williams. Pp. xvi., 359. London: Baillière, Tindall and Cox. 1901.

In this work the author has produced a very important contribution to the pathological study of new growths which affect the female generative organs.

The book is compiled with great care, and with what is almost a superabundance of statistical detail.

In the statistical portion proper some interesting points are brought out, one of which is the excess of carcinomatous neoplasms of the uterus over all others. This the author shows to be very marked, carcinoma of the uterus occurring in a proportion of very nearly 3 to 2 when compared with all other neoplasms of this organ.

The chapters on anatomy and development are very thorough and well illustrated, the illustrations of microscopical preparations being especially clear.

A very interesting chapter is that on the causation of cancer. We fear that in this many of the author's conclusions will not seem quite tenable, since statistical figures as a basis for argument have not that stability which might be wished, and frequently lend themselves equally to a precisely opposite deduction. The author hints that cancer is comparatively infrequent among vegetarians, prisoners, paupers, and lunatics,—in each of these classes the consumption of meat is small,—while the disease is comparatively frequent among the well-to-do and agricultural classes. As a matter of fact the great bulk of the agricultural population consume very little meat.

The chapters on carcinoma and fibroid tumours are especially interesting, and contain a wealth of valuable information.

The enormous amount of information contained in the work makes it almost impossible to compress any indication of its scope into a review. It is a monument of industrious compilation, and a most interesting contribution to the literature of the subject.

